# Computational screening identifies selective aldose reductase inhibitors with strong efficacy and limited off target interactions

**DOI:** 10.1038/s41598-025-12859-x

**Published:** 2025-08-01

**Authors:** Jicli Jose Rojas, Roberto Pestana-Nobles, Leonardo C. Pacheco-Londono, Jesús Utria-Munive, Nataly J. Galan-Freyle

**Affiliations:** 1 Life Science Research Center, Universidad Simon Bolívar, 080002 Barranquilla, Colombia; 2 Grupo de Investigación en Sistemas Autónomos e Inteligencia Artificial, UFOTECH, 080002 Barranquilla, Colombia; 3 Faculty of Basic and Biomedical Sciences, Universidad Simon Bolívar, 080002 Barranquilla, Colombia; 4 Faculty of Medicine, Universidad Simon Bolívar, 080002 Barranquilla, Colombia

**Keywords:** Diabetes mellitus, Aldose reductase, Docking, Molecular dynamics, Inhibitors, Antitarget proteins, Computational biology and bioinformatics, Drug discovery

## Abstract

Diabetes mellitus is characterized by persistent hyperglycemia that triggers micro-vascular complications in organs such as the eyes and kidneys; a pivotal enzymatic driver is aldose reductase (AR), which reduces glucose to sorbitol. Because existing AR inhibitors often cause off-target toxicity, we implemented an integrative in-silico workflow to discover selective, safer compounds. A library of 4 975 small molecules was docked against AR and, in parallel, against five clinically relevant antitarget proteins or proteins whose unintended inhibition is associated with adverse pharmacological or toxicological effects (CYP2A6, CYP2C9, CYP3A4, SULT1A3 and the pregnane X receptor), retaining 236 ligands whose binding energies to every antitarget were weaker than those of the reference drug tolrestat. These survivors were redocked to five high-resolution human AR crystal structures, and the ten best-scoring ligands underwent 100 ns molecular-dynamics simulations followed by MM-PBSA free-energy calculations to refine affinity estimates and probe complex stability. Ligand 4934, a benzo[a]anthracene–pyrene polyphenol, displayed the strongest predicted affinity for while showing poor affinity for the antitarget panel, outperforming tolrestat by more than 2 kcal mol⁻¹ and adopting a stable plug-like pose that occludes the catalytic pocket through extensive π–π and hydrophobic contacts with Trp111, Phe123 and Lys22. These findings highlight ligand 4934 as a promising scaffold for selective AR inhibition and demonstrate the effectiveness of the stepwise computational strategy in prioritizing lead compounds with reduced off-target liabilities.

## Introduction

Diabetes mellitus is a medical condition characterized by hyperglycemia as consequence of body’s inability to use blood sugar for energy^1^. Its exponential growth represents a significant risk to health since it is a high-cost chronic disease with high morbidity and mortality. The number of adult people (20–79 years) with diabetes is 537 million and is expected to reach approximately 783 million diagnosed people by 2045^2^. In 2019, diabetes and kidney disease due to diabetes were mentioned as a cause of death in estimated 2 million deaths^3^.

Diabetes mellitus is a metabolic disorder that occurs by high blood sugar levels in the body and hence the glycolysis is affected. In the normal process, the hexokinase enzyme allows the glucose enter through a pathway called the polyol pathway and two key enzymes present perform its conversion into sorbitol and fructose with the help of aldose reductase (AR) and sorbitol dehydrogenase (SDH), respectively^4^. In chronic diabetes, as AR is found predominantly in tissues like the retina, lens, nerve, vascular, and glomerulus, the polyol pathway plays a crucial role in the development of microvascular complications related to the eyes, nerves, and kidneys, and furthermore, it results in high consumption of NADPH, cofactor utilized by AR to convert glucose into sorbitol^5,6^. The accumulation of sorbitol causes cellular damage due to high osmotic pressure because the conversion of sorbitol to fructose occurs slowly and does not easily cross cell membranes^7^.

NADPH is involved as cofactor in others important metabolic process such as conversion of oxidized glutathione (GSSS) to its reduced form through glutathione reductase enzyme and it is necessary by biosynthesis of fatty acids and nitric oxide (NO). But in diabetes complications, by excessive consumption of NADPH results in a decrease in the content of glutathione. Insufficient glutathione levels initiate a series of oxidative stress reactions^8^.

AR has been extensively studied as a key target in the prevention and management of diabetic complications. The inhibition of AR is considered a promising strategy for preventing or delaying the onset of diabetic complications by inflammation processes. In recent decades, efforts have focused on the search for molecules capable of inhibiting the action of AR. The structure of AR present basically two active site to bind inhibitor molecule, the first one is polar moiety called “anion binding pocked” which is constituted by TYR48, HIS110 and the C4N of the nicotinamide coenzyme, and the other one is a hydrophobic moiety (includes those that form the walls of the hydrophobic cleft — TRP20, TRP79, TRP111, PHE122, PRO218, TRP219, CYS298, LEU300 and VAL47)^9,10^.

**Fig. 1:**
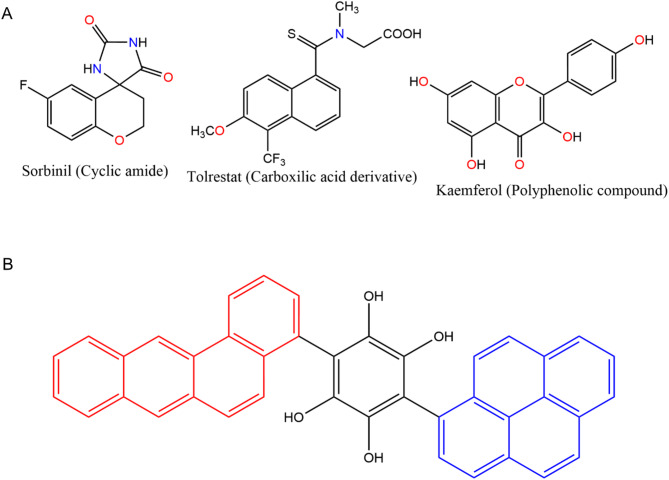
Representative aldose-reductase inhibitors. (**A**) Three well-studied scaffolds illustrate the chemical space historically explored for AR inhibition. Sorbinil is a cyclic amide (a benzofuran-lactam) whose endo-amide carbonyl and exocyclic imide NH groups provide a bidentate hydrogen-bond motif that anchors the inhibitor in the catalytic pocket. Tolrestat is a carboxylic-acid thiochromanonate; its anionic carboxylate forms an ionic/hydrogen-bond network with Tyr48 and His110, while the CF₃ substituent adds lipophilicity that improves passive uptake but also contributes to off-target CYP binding. Kaempferol, a flavonol polyphenol, combines an extended π-conjugated system with four phenolic OH groups; the former promotes π–π stacking with Trp111/Phe123, whereas the latter furnish multiple hydrogen-bond donors/acceptors that enhance affinity but reduce metabolic stability. (**B**) Ligand 4934 (highlighted in red and blue) fuses two polycyclic aromatic systems through a rigid flavone-like linker, merging the hydrophobic cores typical of Tolrestat analogues (red) with the hydrogen-bond-rich catechol terminus characteristic of flavonoids (blue).

Several molecules as AR inhibitor have been synthetized with multiples structures but they have some common feature that allow them to be classified in three main classes: compounds that contains cyclic imides, carboxylic-acid derivatives and polyphenolic compounds^11^ (Fig. 1A).

There are a variety of AR inhibitor belonging to the class of carboxylic acid derivatives such as Alrestatin, Tolrestat and IDD594 that show a great affinity for the enzyme in vitro but their potency is highly reduced in vivo. The decreased efficacy observed in vivo can be attributed to the low pKa of these inhibitors, resulting in ionization at physiological pH levels. As a consequence of the negative charge, these compounds face difficulty to pass through the cell membrane^11^.

Carboxylic-acid aldose-reductase inhibitors such as Alrestatin, Tolrestat, and IDD594 bind the enzyme with high affinity in vitro, yet their pharmacological potency drops sharply in vivo because their low pKₐ values leave them predominantly ionized at physiological pH; the resulting negative charge severely limits passive diffusion across cell membranes, reducing intracellular concentrations and therapeutic effect^11–13^.

Tolrestat is the measurement pattern in this study since despite it has low permeability and leads to health problems^14^it is a potent AR inhibitor^10,15,16^ confirmed by crystal structure determination of the complex that elucidates its binding characteristics^17^.

AR inhibitors often produce *significant side effects* due to interactions with off-target proteins, commonly referred as antitarget, these are proteins whose unintended inhibition is associated with adverse pharmacological or toxicological effects^18,19^.

An antitarget is a protein or molecule that a potential drug should avoid binding in order to prevent harmful side effects. The failures found in the first AR inhibitors are not only associated with their effectiveness in treating diabetes complications, but also with the side effects they produced^20^. This side effects are usually associated with interaction of this antitarget proteins. Among possbiles antitarget proteins the Cytochrome P450 (CYP P450s) can be considered a good antitarget, playing an important role in the metabolism of a wide range of compounds, including drugs, toxins, and endogenous substances. If a CYP enzyme is inhibited by a compound its enzymatic activity may be reduced^21^. Similarly, the sulfotransferases (SULTs) which catalyzed reactions in the phase II metabolism which take the products from enzymes phase I metabolism, like CYP P450s, attach them a sulfonyl group and making them easier to excrete or to avoid being link to others receptors^21^. The pregnane X receptor (PXR) is a transcription factor that regulates the gene expression of a variety of phase I and phase II enzymes including CYP P450s, SULTs, and drug transporters. PXR has shown the ability to bind to a wide variety of different compounds^21^ making it a good antitarget.

In drug discovery, computational techniques, particularly molecular docking and molecular dynamics simulations, have been widely employed for the rational design and optimization of drug candidates. Numerous studies have been conducted using these methods^22–27^yielding interesting results. Therefore, this study through molecular docking and molecular dynamics addressed the search of novel AR inhibitors, 4975 ligands were evaluated against 5 antitarget proteins and the protein AR, the best ligands were evaluated through molecular dynamics simulations and MM-PBSA calculations.

## Results and discussions

**Fig. 2:**
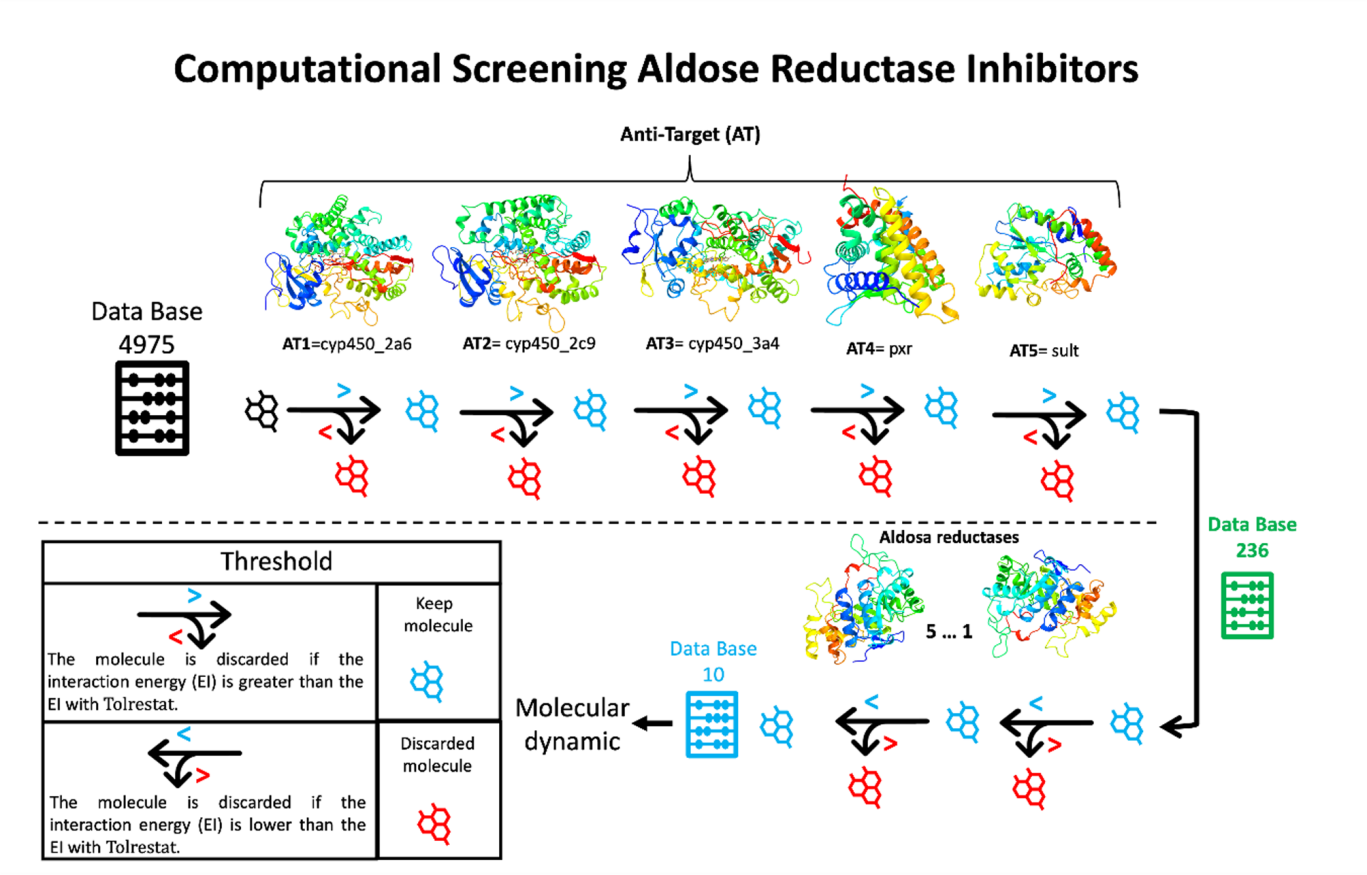
Workflow scheme of the whole computational screening of aldose reductase inhibitors. Initially all 4975 compounds were screened sequentially against each of the antitarget protein. The best 236 ones were dock against Aldose Reductases (AR) proteins, where the best 10 compounds were evaluated through molecular dynamics and MMPBSA calculations.

The Fig. 2 shows the workflow of molecular docking process. The first stage, Molecular docking part I, of this study was to identify if any molecule of our set of ligands has a better interaction than Tolrestat when is screened against antitarget protein’s binding site by molecular docking, and those molecules are considered to cause side effects or toxicity. Based on the 24,875 scores obtained from 4975 ligands and 5 antitarget proteins (CYP450s 2a6, 2c9 and 3a4, SULT1A3 and PXR), the script presented from code availability was applied in order to analyze these results. The initial step of the script takes all docking results obtained from the first protein (labeled AT1 in Fig. 2), discards those where ligand-protein interaction energy (IE) is greater than IE of tolrestat-AT1 complex, and considers the rest ligands to be analyzed using their results from the next protein (AT2). This step is repeated until involve all protein-docking results (AT1…AT5). At the end, the application of the script showed that 236 ligands have lower IE than tolrestat when they were tested with antitargets protein and they were considered to be tested in molecular docking against AR protein (5…1 in the Fig. 2) in the next stage, Molecular docking part II.

### Molecular docking part I: antitarget proteins

The molecular docking results of CYP P450 2a6 protein showed the lowest scores among the tested antitarget proteins, which can be attributed to its small volume and that its active site cavity is less than 25% of the volumes calculated for structures of the human drug-metabolizing 2c9 (PDB ID: 1R9O) and 3A4 (PDB ID: 1TQN)^28,29^. Otherwise the ligands’ docking results with P450 2c9 and P450 3a4 present scores closer to tolrestat’s score which can be explained by large cavity binding site with important polar and hydrophobic interactions^30–32^ and remarkable flexibility leading to high promiscuity^33^respectively.

**Fig. 3:**
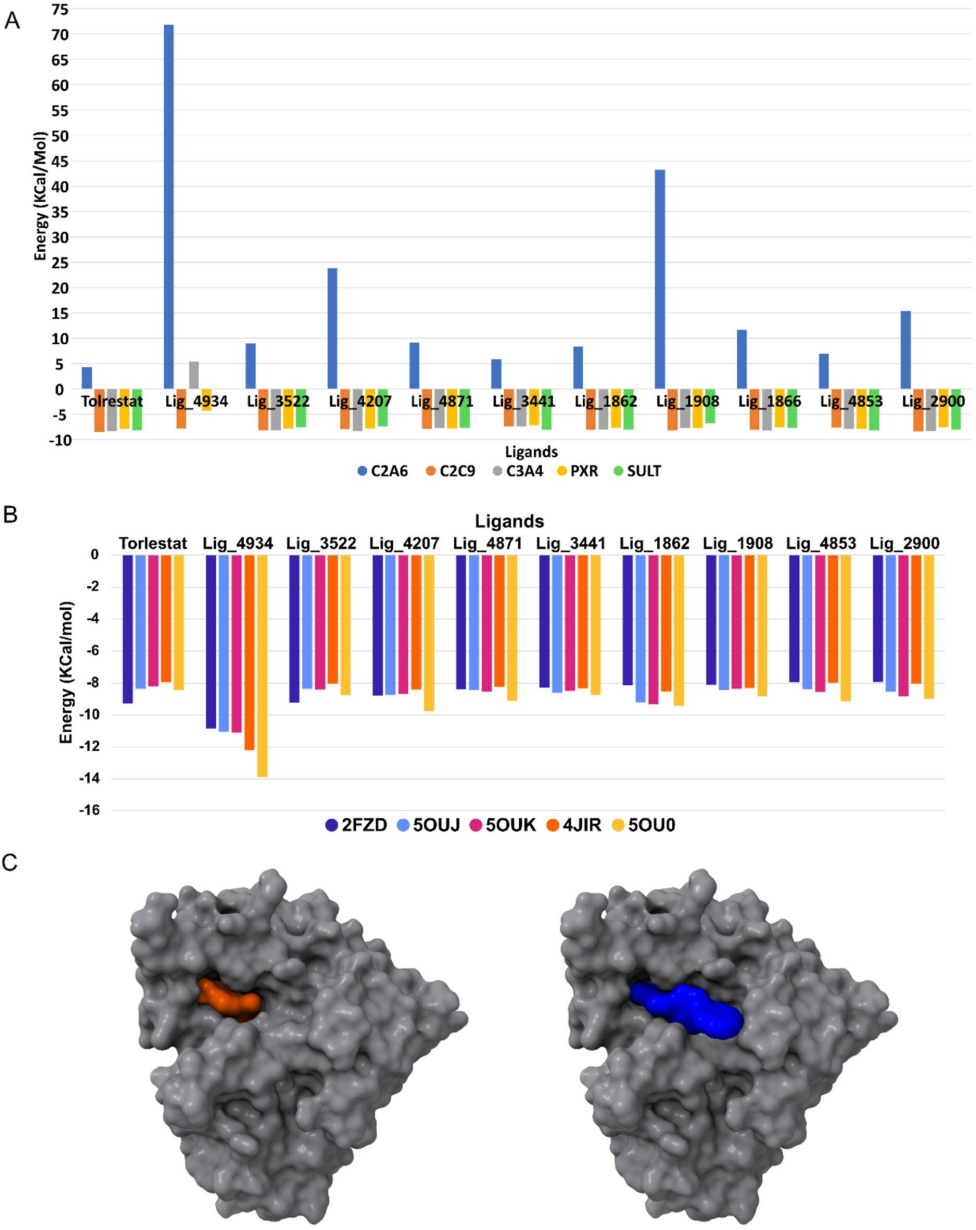
**(A)** Anti-target docking results for ligand the best 10 ligands, the energy of SULT-4934 complex is too close to zero to be see observe in the chart. **(B)** Docking results against the AR proteins showing the best 10 ligands. **(C)** Surface maps of complex AR-tolrestat and AR-4934 from ChimeraX v1.8.

**Fig. 4:**
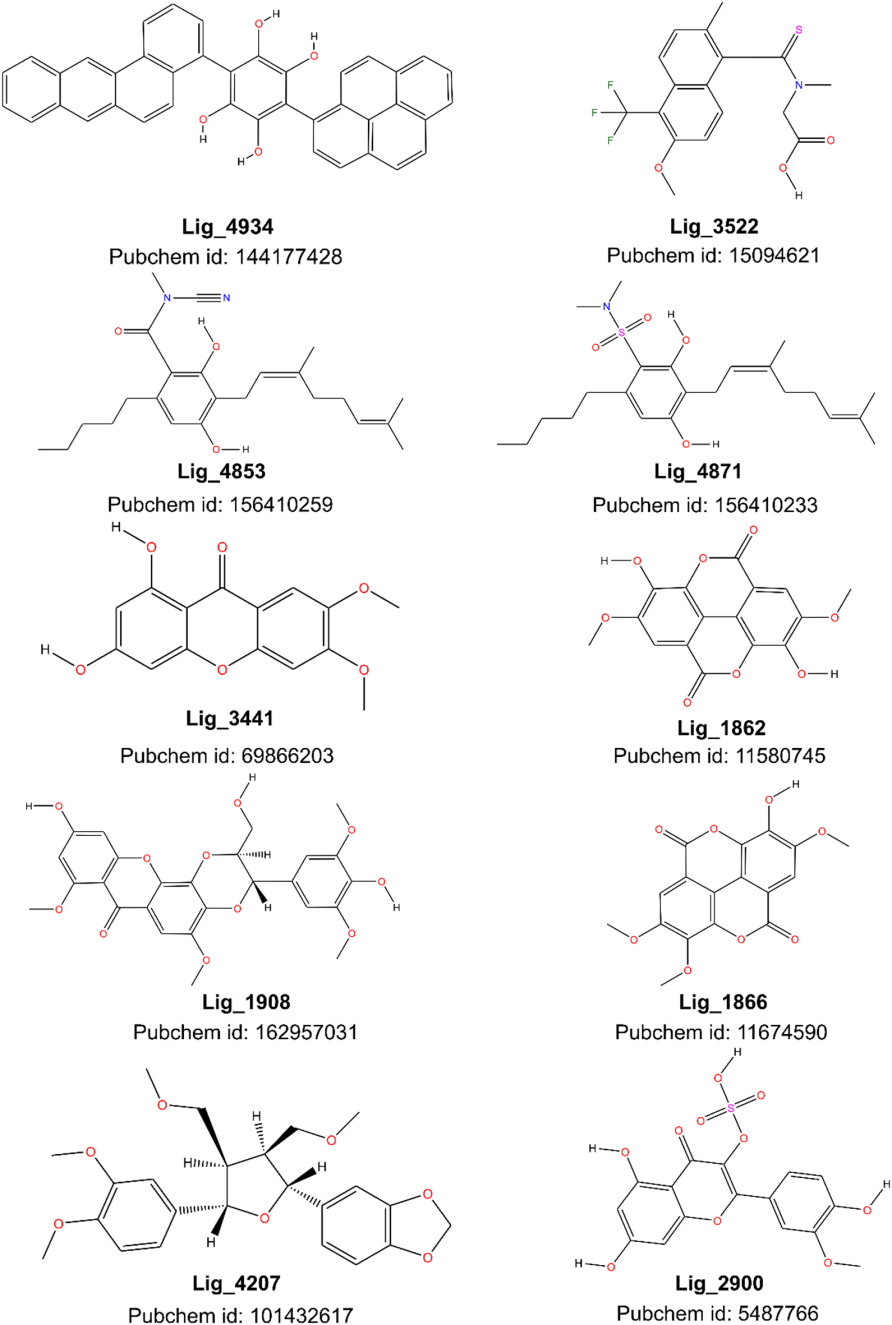
The structures of the best 10 ligands considered in this study, including the Pubchem ID of each of them.

The scoring results obtained from the docking performed with protein SULT1A3 (the crystal used in this study) are similar to CYP P450s 2a6 and 3a4. The affinity can be attributed to the active site of SULT1A3 that involves several residues, including HIS108, LYS106, GLU146, ASP86, and residues from the PSB-loop (TYR45, PRO46, LYS47, SER48, GLY49, THR50, and THR52). These residues are involved in hydrogen bond interactions and stacking interactions with the substrate and cofactor molecules^34^. PXR is known to adapt its binding pocket to chemically diverse ligands in crystallo; although our rigid-receptor docking cannot capture this induced-fit process explicitly, the pocket’s large cavity and documented conformational plasticity help explain the higher docking scores observed^35^. Figure 3A show the best 10 ligands, which got the best interaction energy with the antitarget proteins, where ligand 4934 (Lig_4934, see Fig. 1B) had the lowest binding interaction among all the ligands in four out of the five antitarget proteins. The structures of these 10 ligands are represented in Fig. 4.

### Molecular docking part II: aldose reductase AR

**Fig. 5:**
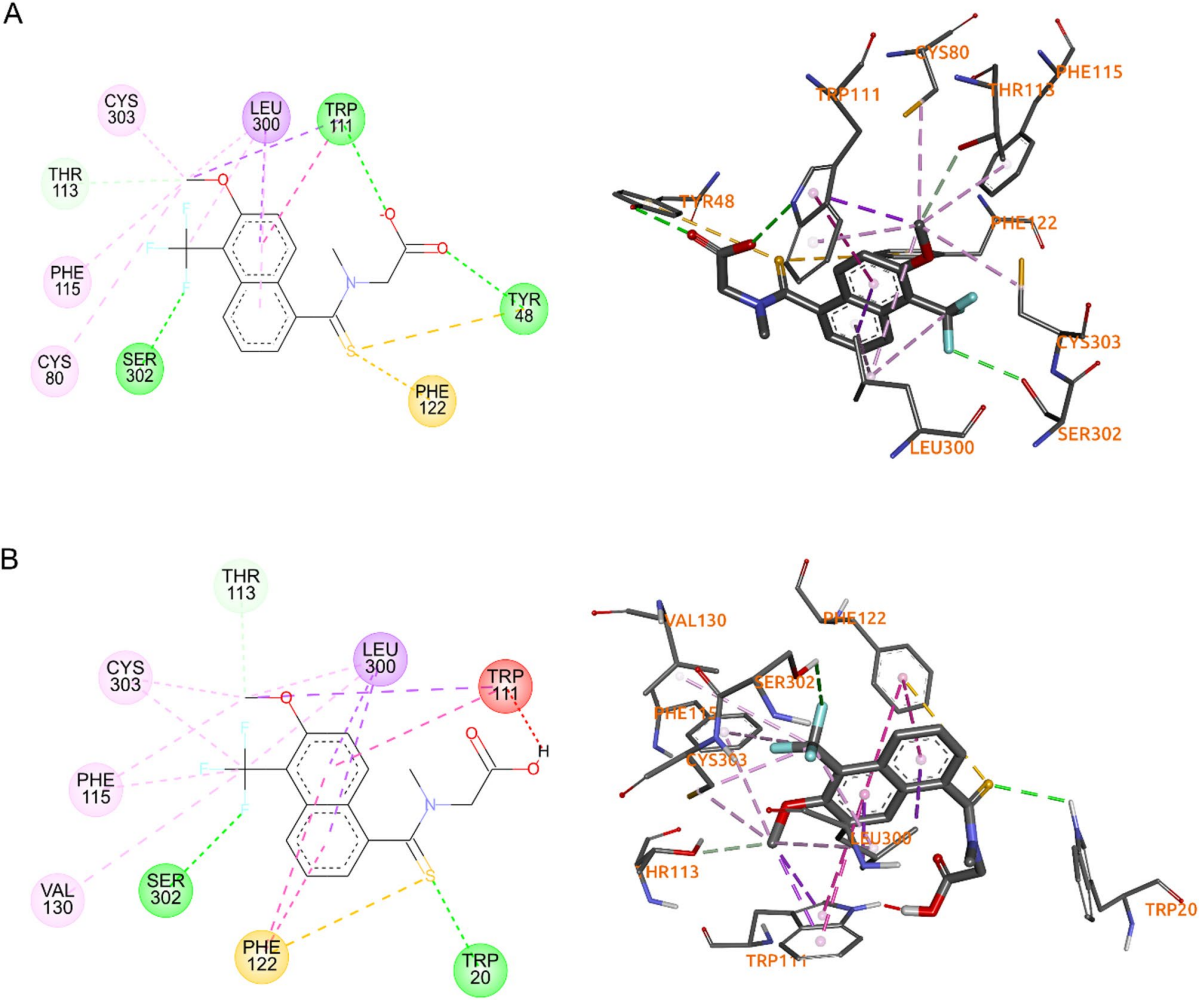
Binding interactions in the native crystal vs. the docking results. **(A)** 2DFZ and torlestat native. **(B)** 2DFZ and torlestat docking results.

The 2DFZ native AR crystal of Tolrestat was selected as a pattern to do molecular docking in this stage. To validate the molecular docking method, Tolrestat was taken it as a ligand to be tested against the same AR crystal 2FDZ and the binding interactions and poses were compared with the native crystal. The docking results were analyzed using Discovery Studio Visualizer, which showed that Tolrestat reaches the active site (Fig. 5) and it has several binding interaction but has the same binding interactions showed in the crystal with the residue TRP111 in the rigid region so-called “anion-binding pocket” and the residues PHE122 and CYS303 in the greater flexibility region called “specificity pocket”^11,17^. The carboxylate group of Tolrestat forms a hydrogen bonds to the backbone NH groups of the side chain indole contained in the TRP111, the sulfoxide group interacts with PHE122 through pi-sulfur interaction and the methoxy group to establish hydrogen bond with CYS303.

Once the Tolrestat was docked against its own native crystal and the method was validated, the 236 ligands were docked against AR protein from crystal 2DFZ and surprisingly the results showed that Lig_4934 got the best binding interaction score and it has a significant difference in values compared to the second one (Tolrestat). Taking into account that Lig_4934 got good results when it was tested against antitarget proteins being the ligand with lowest interaction, we decided to replicate molecular docking using others four different AR crystals, 5OUJ, 5OUK and 5OU0, which have 1-oxopyrimido[4,5-c]quinoline-2-acetic acid derivatives as an inhibitor, larger molecules than Tolrestat, and JIR4 AR crystal with Epalrestat as an inhibitor slightly more flexible than Tolrestat. The Fig. 3B showed the scores of the best 10 results from these AR proteins and the calculations confirm that Lig_4934 has the best binding interaction with AR protein.

**Fig. 6:**
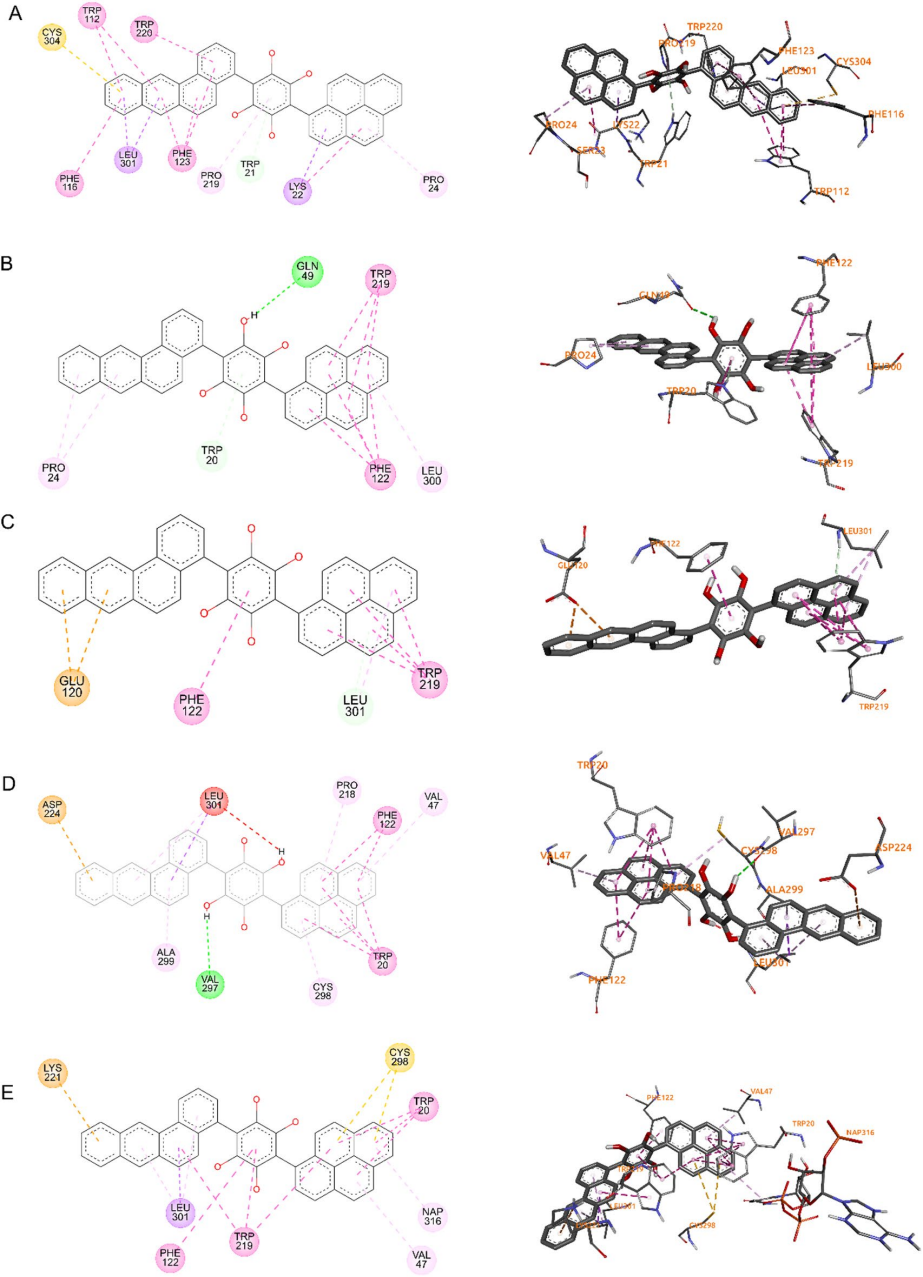
Three-dimensional 3D and two-dimensional 2D structure of lig_4934 with 5 AR protein structures. **(A)** 2FZD, **(B)** 5OUJ, **(C)** 5OUK, **(D)** 5OU0, **(E)** 4JIR.

To explain the molecular behavior of Lig_4934, both complex AR-Lig_4934 and AR-Tolrestat were compare through surface maps, and the image in Fig. 3C shows that Tolrestat occupied the active site into the pocked while Lig_4934 acts as a plug, blocking the entrance to the active site. To describe binding interactions, the Fig. 6 show the three-dimensional 3D and two-dimensional 2D structure of AR target proteins in complex with Lig_4934 (2DFZ, 5OUJ, 5OUK, 5OU0 and 4JIR). It can see that Lig_4934 has two aromatic systems with four fused rings attached to a polyphenol ring in a perpendicular manner. In the complex 2DFZ, the Lig_4934 is link to the protein through interactions that involve pi bonds of aromatic rings and one of the aromatic system, benzo[a]anthracene unit, is accommodated within the active site pocked with interactions over its two faces with the residues TRP111, TRP219, CYS303, PHE115, LEU300, PHE122, while the others two aromatic systems, pyrene and polyphenol units, cover the entrance to the active site, the pyrene unit interacts with residues LYS21 and PRO23 over only one face of rings system and the polyphenol ring interacts with residue PRO212.

The results of the molecular docking of Lig_4934 with the other crystals show similar poses and the majority of binding interactions involve pi bond as well, only crystal 5OU0 and 5OUJ showed conventional hydrogen bond with the hydroxy group of the polyphenol unit.

### MD simulation

**Fig. 7:**
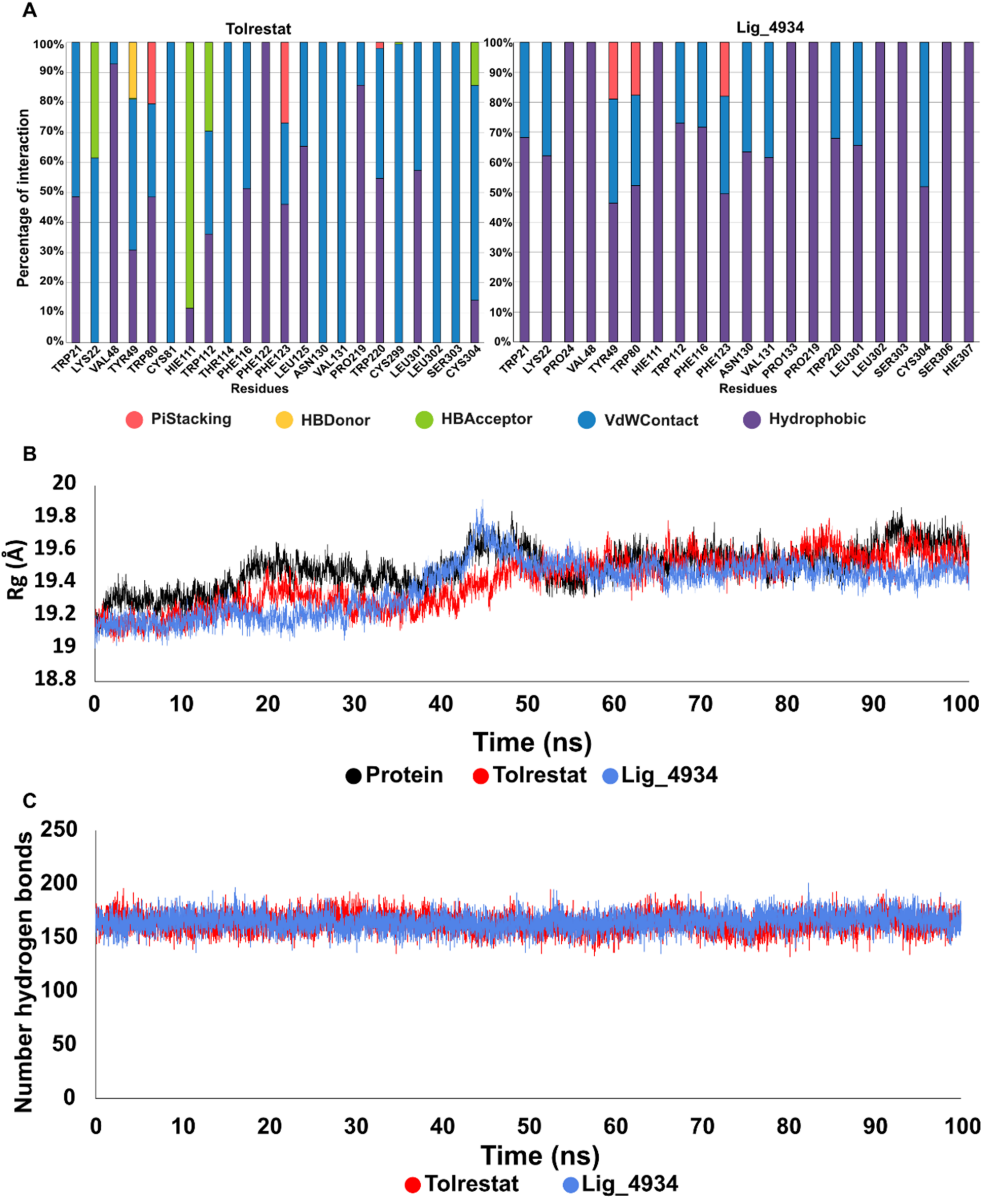
Comparing different measures between Tolrestat and ligand 4934 (Lig_4934). **(A)** Type of interaction, in the left Tolrestat, in the right Lig_4934. **(B)** Radius of gyration, showing the protein in black, protein-torlestat in red and protein-Lig_4934 in blue. **(C)** Number of hydrogen bonds present during the simulation, in red protein-Tolrestat, in blue protein-Lig_4934.

**Fig. 8:**
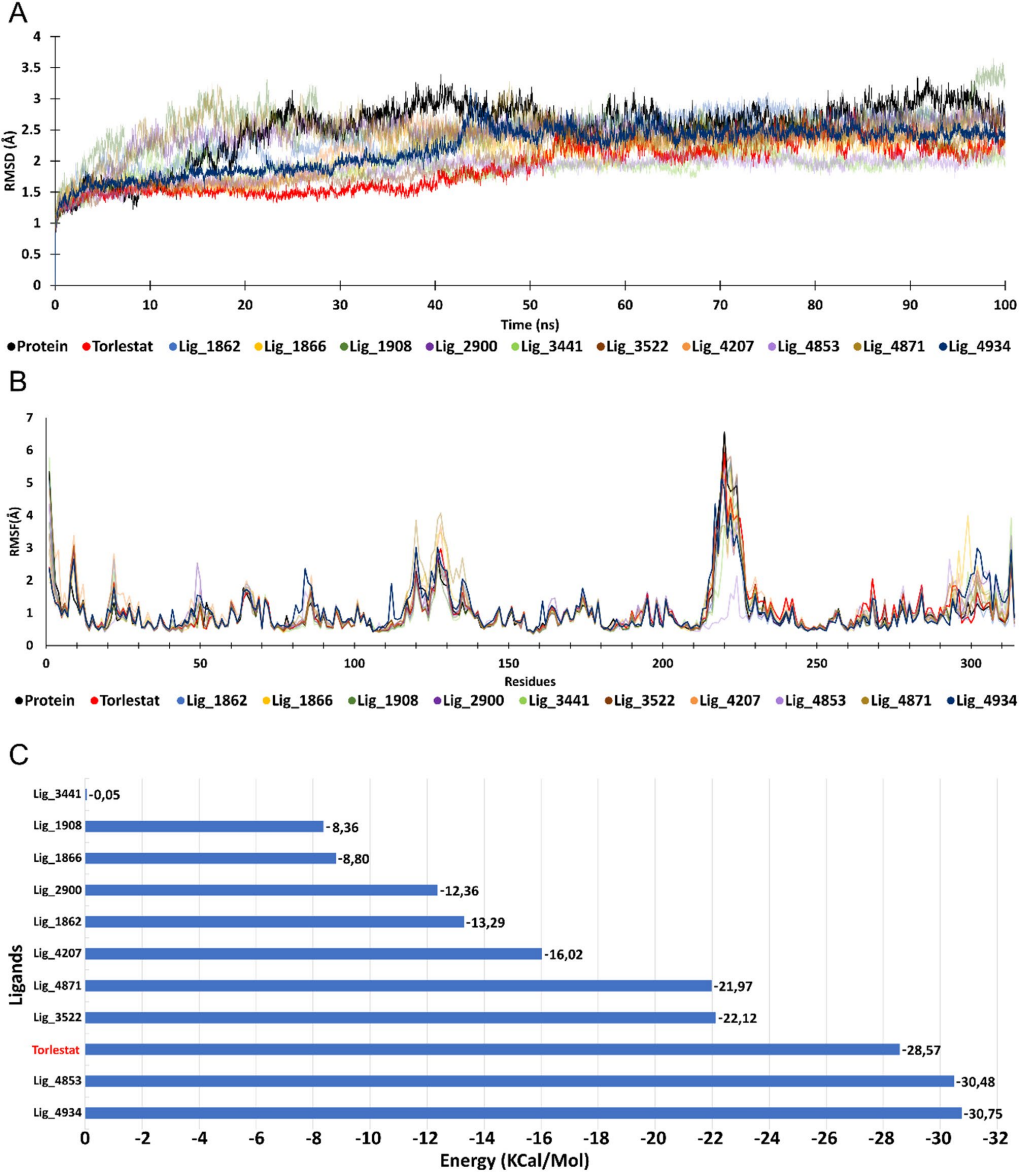
**(A)** RMSD deviation of AR-4934, AR-Tolrestat complexes and AR uncomplex. **(B)** RMSF. **(C)** MMPBSA. RMSD and RMSF highlight the result for Lig_4934 because is the best ligands among the all analyzed. The MD trajectory indicates that the mobility of the complex is directly related to local structural changes as is showed in the Fig. 9. The Figs. 8B and 9A correspond to a frame 1 and 10,000 of the simulation, respectively, the green area in Fig. 8A shows the residues that are contact with ligand within a distance of 6 Å and the same color area in the Fig. 9B shows the same residues but in the end state with another conformation. Per-residue MM-PBSA decomposition shows that hydrophobic and π–π stacking contacts are the main stabilising forces for the complex, complemented by a persistent hydrogen bond between the ligand carbonyl and Tyr48; no long-lived salt bridges were observed throughout the simulatior.

The result of molecular docking showed that Lig_4934 acts as a plug blocking the binding cavity of AR enzyme through pi-interactions mostly. To validate docking results of the complex AR-4934, MD simulation was performed to the top 10 docking results for 100 ns. MD trajectory generated from AMBER suite programs was analyze using MMPBSA method to calculate the binding free energy for the association between components of the complex, in the case of AR-4934 complex got the highest binding free energy (Fig. 8C) supporting the results obtained in the molecular docking as the best affinity result. To confirm what kind of binding interaction is present between Lig_4934 and specific residues of the protein and the time interaction during MD simulation, Python ProLIF^36^ library was used and the information is summarized in the Table 1 and the Fig. 7A. The information extracted shows that in the AR-4934 complex, hydrophobic is the predominant binding interactions, being TRP21, PHE116, PHE123, VAL131 TYR49 and LYS22 residues that present percentage of time contact greater than 50%. When Tolrestat is used to compare this information, the interactions it presents, for the most part, are of Van der Walls interactions being the presence of hydrogen bonds important but they have established binding interactions with the same residues in the hydrophobic moiety (Table 1). Figure 7B compare the radius of gyration (Rg) among the protein alone and the protein bound to Tolrestat and Lig_4934, showing that neither Tolrestat or Lig_4934 alter the protein structure and stability. Figure 7C show the number of hydrogens bonds in the system without considering possibles bond with the solvent, both Tolrestat and Lig_4934 show similar number of hydrogens bonds during the entire simulation, but when we analyzed the representation of hydrogen bond between protein and ligand, this are not present more than 5% of the simulation, meaning that the interaction between protein and ligand in this case, for both ligand comes from other types of interaction like VdWContact and Hydrophobic interaction, like Fig. 7A shows.

**Table 1:** Residue with an interaction greater than 30% and percentage of time interaction, bold residues make interaction with Tolrestat and lig_4934.

Ligand	Residues
Tolrestat	**LEU301(99.91%)**, **PHE123(99.59%)**, TRP80(99.55%), **TRP112(98.75%)**, HIE111(93.27%), VAL48(78.42%), CYS304(77.44%), **TRP21(73.77%)**, **TYR49(72.35%)**, **PHE116(69.33%)**, THR114(40.02%)
Lig_4934	**PHE123(97.08%)**, **PHE116(95.04%)**, **TRP21(86.74%)**, **TYR49(73.51%)**, LYS22(73.31%), VAL131(72.37), ASN130(69.88%), SER303(53.91%), **LEU301(44.92%)**, LEU302(40.82%), SER306(38.16%), **TRP112(35.74%)**, TRP80(30.10%)

The Fig. 8A and B show the mobility behavior of AR-4934, AR-Tolrestat complexes and the uncomplex AR based of RMS deviation and RMS fluctuation respectively, from X-ray structure used to build the systems. The trajectory average seen in the RMSD result is 2.20 ± 0.35 Å, 1.89 ± 0.37 Å and 2.52 ± 0.47 Å respectively, this result shows a better stability of AR-Tolrestat complex during simulation time and at the first five thousand frame it seems to be a period where Tolrestat and Lig_4934 spend time to fit binding site cavity, after this time the complex reach the stability. additionally, the results of RMS fluctuations, indicate an important residual mobility between the LEU301 and TYR310 side chains in the AR-Lig_4934 complex as a consequence of the change of contact from residue LEU301 in the early phase of the simulation with hydrophobic interactions between its side chain isobutyl group with the benzo[a]anthracene unit of Lig_4934 to SER303 at the end of simulation with a hydrogen bond between the oxygen of hydroxy group of SER303 and the hydrogen of the hydroxy group of polyphenol unit of Lig_4934.

**Fig. 9:**
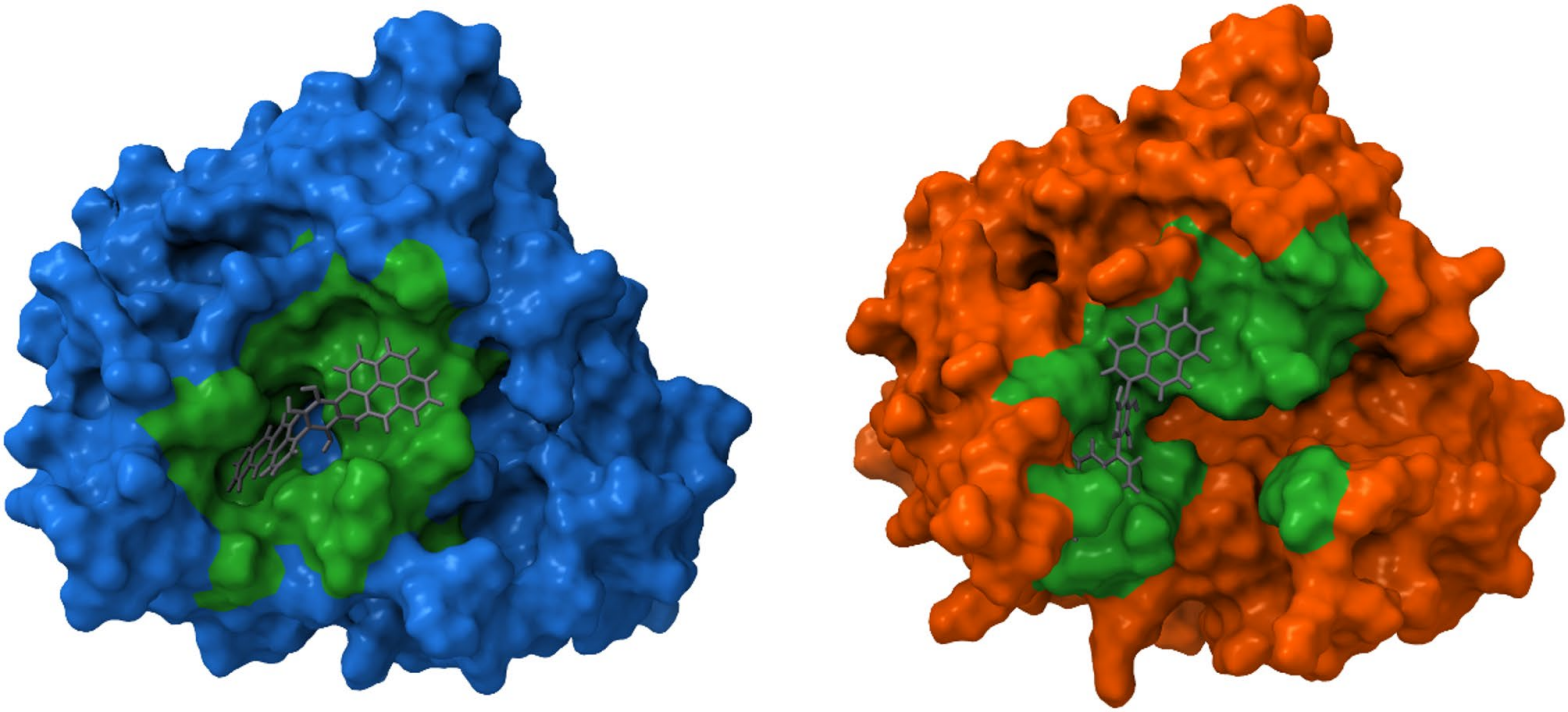
**(A)** Frame 1 of AR-4934 completed MD simulation from ChimeraX v1.8. **(B)** Frame 10,000 of AR-4934 completed MD simulation from ChimeraX v1.8.

## Materials and methods

### Proteins preparation

The set of protein targets and antitargets were obtained from the RCSB Protein Data Bank (RCSB PDB)^37^. The protein targets included the structure of Human aldose reductase 2FZD^17^ complexed with Tolrestat, the drug reference of this study. Tolrestat is potent AKR1B1 inhibitor of the class of carboxylic acid derivatives with the ability to improve neuropathy and nephropathy in diabetic complications^10,11,38^. Other protein targets were chosen as representative complexes from different crystallized AKR1B1 enzymes (5OU0^39^, 5OUJ^39^5OUK^39^ and 4JIR^40^resolutions among 0,94 and 2,0 Å) taking them as the template for docking to stablish comparison with docking results from crystal structure 2FZD and to improve the reliability of the results.

The antitargets set contained the human sulfotransferase 1A3, pregnane X receptor, and three cytochrome P450 enzymes (2a6, 2c9 and 3a4). For each protein, all water molecules and any other solvent in the PDB file was removed using Chimerax. All hydrogen were removed. In case of missing atoms PDBFixer 1.9 was used, available at (https://github.com/openmm/pdbfixer; accessed on 16 July 2023). Molecular docking was conducted to predict the interactions between these enzymes and ligands, which in turn can provide insight into potential side effects and toxicity. By improving selectivity, this approach can help optimize drug discovery and development.

### Ligands preparation

All ligands were found from PubChem (https://pubchem.ncbi.nlm.nih.gov) chemical information resource, developed by the US National Institutes of Health^41^. The selection of each ligand structure was based on the search for compounds that are structurally similar to commercial drugs on the market against to diabetes complications, using the premise that “similar structures are likely to have similar biological activities”. A total of 4975 potential ligands were found and docked against targets and antitargets proteins.

For docking, each ligand was prepared using MEKKO, a python library which add polar hydrogen in case of being absent and calculate Gasteiger charges, at the end, the library gives you the pdbqt file for the docking.

### Antitargets and targets-protein–ligand dockings

Docking of the 4 975 ligands—including Tolrestat as reference—against both antitarget and target proteins was performed with AutoDock Vina 1.2.3^42^ using the Vina scoring function. Structures were converted to PDBQT with AutoDock Tools4^42^: only polar hydrogens were added and Gasteiger charges assigned. For each antitarget, a 40 Å cubic grid was centred on the catalytic pocket to enclose the entire active site; for the human aldose-reductase structures, a blind-docking box covering the whole protein was defined. No induced-fit or side-chain optimisation algorithm was applied, so receptor backbones and side-chains were kept rigid during docking. Vina was then executed, and the pose with the most negative binding energy was retained for every complex. All ligands were first docked to the antitargets; compounds exhibiting stronger affinity than Tolrestat were discarded to minimise potential off-target toxicity. Only ligands with weaker antitarget binding than Tolrestat were advanced to docking against the selected human aldose-reductase proteins.

### Targets-protein–ligand molecular dynamics (MD) simulation

Molecular dynamics was made using Amber22^43^ suite of biomolecular simulation programs.

### System Preparation

The force field used to protein was ff19SB^44^ the most current protein force field. The ligand parametrization for all compounds was made using GAFF2^45^ force field which was updated from GAFF to improve bonded parameters to reproduce molecular geometries. The parameters for the cofactor NADP + were taken from the parameters established by Ryde^46^. Resp charges were calculated for each ligands using antechamber taking electrostatic potential data generated from gas-phase calculation using Gaussian 16 program^47^ at the B3LYP/6-311 level of theory, considering dispersion correction g3bj. For this process, the conformational structure of all ligands was obtained from previous results in molecular docking and the hydrogen atoms were placed using Open Babel^48^ chemistry toolbox.

The protein crystal structure 2FZD was taking to make MD study, consists in 313 amino acids put together to the cofactor and the ligand. The entire system (protein-ligand) was solvated in a box, loaded with water molecules using TIP3P water model (box size 79.1 Å x 68.4 Å x 80.8 Å and total 10049 water molecules), finally, Na + or Cl- ion was added to neutralize the system.

### Molecular dynamics (MD)

The first step of MD simulations was the minimization of the water molecules leaving the solute fixed, using a constant force of 100 kcal/mol- Å^2^following the system minimization using 1000 cycles with steepest descent out of 100,000 total cycles. Then, the solvent was heated using a NVT ensemble to 300 K over a period of 0,5 ns keeping the solute fixed with a force of 2 kcal/mol- Å^2^. After this procedure, pressure was adjusted carrying out 50 ps of simulation applying a NPT ensamble and periodic boundary conditions with constrain in all bonds involving hydrogen atoms (constrained by the SHAKE algorithm^49^ and under constant pressure and temperature (The Langevin dynamics was applied with the collision frequency 2 ps-1 given in gamma_ln). A cutoff of 8 Å was applied to the van der Waals. Then, after 0.5 nanoseconds, the system reached an equilibrium state with respect to its total potential energy and the production simulation was run for 100 ns. All dynamic process was done with the pmemd.cuda MD module of Amber 22 and VMD was used to visualize the results.

### MM-PBSA

Binding free energy was calculated using the MM-PBSA script from Amber package^43^. For this calculation, only the last 2 ns of the simulation were considered, taking a snapshot each 0.01 ns, giving a sample of 200 snapshot. Entropy was not considered due to the high computational cost and the potential to reduce the accuracy of the calculation^50,51^. The parameters were set as follows: inp = 1 and radiopt = 0, and the energy decomposition was enabled by setting the idecomp option to 1.

### Protein-ligand interaction

In order to get protein-ligand interaction, python library ProLIF was used, for this the entire simulation was considered (100 ns) taking a sample each 0.01 ns.

## Conclusion

Diabetes mellitus can become a major preoccupation worldwide considering how much is rising the cases year by year, different strategies are being taken to tackle this disease, in this study through molecular docking and molecular dynamics, a total of 4975 compounds were analyzed among different crystal for the aldose reductase protein in order to find a possible new candidate to inhibit the aldose reductase. The results of this study propose one ligand (Lig_4934) that can be consider as a inhibitor for aldose reducatse, Molecular dynamics simulation show that VdwContact and hydrophobic interaction and the most predominant for the interaction with this ligand, and the MMPBSA calculation report a better binding compared to the Torlestat, which is the ligand used as reference in this study. The ligand 4934 (see Fig. 1B) is a good candidate for a new starting point for other in silico strategies as lead optimization to propose new candidates using this ligand as template and in this way improve its activity.

## Data Availability

The datasets generated and/or analyzed during the current study are available from the corresponding author on reasonable request.

## Code availability

The scripts used in this study are availability from https://github.com/JicliJose/Filter_antitarget-.
